# Reliable, resilient and sustainable water management: the Safe & SuRe approach

**DOI:** 10.1002/gch2.1010

**Published:** 2016-06-17

**Authors:** David Butler, Sarah Ward, Chris Sweetapple, Maryam Astaraie‐Imani, Kegong Diao, Raziyeh Farmani, Guangtao Fu

**Affiliations:** ^1^ Centre for Water Systems, College of Engineering, Mathematics and Physical Sciences University of Exeter Exeter UK; ^2^ Department of Engineering and the Built Environment, Faculty of Science and Technology Anglia Ruskin University Chelmsford UK

**Keywords:** framework, interventions, reliability, resilience, sustainability, water management

## Abstract

Global threats such as climate change, population growth, and rapid urbanization pose a huge future challenge to water management, and, to ensure the ongoing reliability, resilience and sustainability of service provision, a paradigm shift is required. This paper presents an overarching framework that supports the development of strategies for reliable provision of services while explicitly addressing the need for greater resilience to emerging threats, leading to more sustainable solutions. The framework logically relates global threats, the water system (in its broadest sense), impacts on system performance, and social, economic, and environmental consequences. It identifies multiple opportunities for intervention, illustrating how mitigation, adaptation, coping, and learning each address different elements of the framework. This provides greater clarity to decision makers and will enable better informed choices to be made. The framework facilitates four types of analysis and evaluation to support the development of reliable, resilient, and sustainable solutions: “top‐down,” “bottom‐up,” “middle based,” and “circular” and provides a clear, visual representation of how/when each may be used. In particular, the potential benefits of a middle‐based analysis, which focuses on system failure modes and their impacts and enables the effects of unknown threats to be accounted for, are highlighted. The disparate themes of reliability, resilience and sustainability are also logically integrated and their relationships explored in terms of properties and performance. Although these latter two terms are often conflated in resilience and sustainability metrics, the argument is made in this work that the performance of a reliable, resilient, or sustainable system must be distinguished from the properties that enable this performance to be achieved.

## Introduction

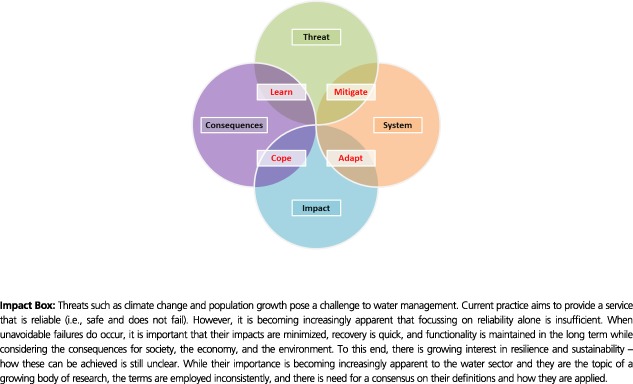

Few would argue with the view that the coming decades will see major challenges to the management of water in cities worldwide, with existing water infrastructure subject to many emerging threats, including climate change, urbanization, asset deterioration, limited resources, and tightening regulation. While currently used methods may be able to cope with future threats individually, water management will become more difficult under highly variable future scenarios, with numerous interconnected global stressors affecting the quality, quantity, and availability of water worldwide (Zimmerman et al. 2008). Recent experiences have revealed serious problems (Hamilton 2009), and levels of service will be significantly challenged by multiple threats and the speed, magnitude, and uncertainty of future change without new ideas and approaches (Butler et al. 2014). Conventional planning and development relies on the ability to project future change (Gleick 2000) and focuses on technical solutions to well‐defined problems (Pahl‐Wostl et al. 2011). However, to successfully address contemporary challenges and provide sustainable urban water management, a transition or shift in paradigm is required (Pahl‐Wostl et al. 2011; Brown and Farrelly 2009).

There has been some gradual change in approaches to water management, with increasing research into resilience (e.g., Lansey 2012 2012; Mugume et al. 2015b) and adaptations (e.g., Haasnoot et al. 2013) for example, but change in practice and actualization of these ideas has been slower. The World Health Organization has recognized the importance of water supply and sanitation resilience in the face of climate change in their 2030 vision (WHO and DFID 2009), and the resilience of water resources has been a focus of the IPCC (IPCC 2001). The United Nations also recognizes that improved water management is critical to ensuring sustainable development and have recommended building long‐term resilience through stronger institutions and investment in infrastructure (UN‐Water 2010). However, the goal of sustainable urban water management seems as far away as ever (Marlow et al. 2013). Furthermore, contestations over the operationalization of resilience mean actions in the international water sector are limited to date (Lansey 2012 2012; Ferguson et al. 2013; Hodbod and Adger 2014; Gearey 2015). Several resilience frameworks do exist in the literature (e.g., Cimellaro et al. 2010; Francis and Bekera 2014; Balica, and Gourbesville 2014; Labaka et al. 2016); however, there are inconsistencies in their approaches to resilience analysis, some measure properties not performance, and they are not typically widely transferable without amendment. These factors are likely to act as a barrier to their implementation, and there is a need for an overarching framework providing greater clarity, consistency, and applicability.

The road to sustainability has been conceptualized as a journey (Butler and Davies 2010); however, every successful journey needs a map and a route to be followed, with stopping off points along the way and an estimate of how much further there is to go. Indeed, there have been recent calls for such an approach (Minsker et al. 2015). This paper aims to provide such a “map” consisting of a set of basic definitions and concepts, a logical evaluation framework, and intervention strategies, enabling water problems and challenges to be addressed in a holistic manner (as recommended by the United Nations (2006)). These provide a logical foundation for analysis of reliability, resilience and sustainability, enabling greater consistency in assessment methodologies and methodical identification of opportunities for intervention. To ensure that they meet the needs of stakeholders, these are produced in collaboration with a range of practitioners and policymakers (including the Welsh Government, the Environment Agency, the Consumer Council for Water, and the Environmental Sustainability Knowledge Transfer Network).

The framework presented supports the development of design and operational strategies for reliable provision of services while explicitly addressing the need for greater resilience to emerging threats, thus leading to more sustainable solutions. It is framed here in the context of water management, with provided examples including water supply, wastewater treatment, urban wastewater systems, and flood management applications. However, the concepts and framework presented could be applied to many different systems.

## Reliability, Resilience and Sustainability

### Definitions

The reliability of a system is typically considered to be its probability of successful operation (Jung et al. 2014) and is defined explicitly in this work as “the degree to which the system minimises level of service failure frequency over its design life when subject to standard loading” (Butler et al. 2014). To measure reliability, the chosen level of service measure(s) and corresponding acceptable limit(s) must be specified. These could be, for example, wastewater treatment plant effluent concentrations and the permitted discharge limits.

There has been much debate over the definition, implementation, and evaluation of resilience since the seminal work of Hashimoto et al. (1982). Resilience has many subtly different definitions (Francis and Bekera 2014) and has been elaborated upon in social, technical, and socio‐technical frameworks (e.g., Wong and Brown 2009; Woods 2015; Schoen et al. 2015). Fiksel (2003), for example, considers a resilient system one that is able to survive large perturbations. This paper uses the resilience definition of Butler et al. (2014), “the degree to which the system minimises level of service failure magnitude and duration over its design life when subject to exceptional conditions”; essentially, it is a measure of how the system performs when subject to unexpected threats that exceed design conditions and the system is unable to meet the required level of service. Alternative definitions include the need for rapid recovery (Jones and Schmitz 2009) and prescribe specific capabilities that a resilient system should possess, such as the ability to adapt and learn (Folke et al. 2002). However, these all aim to reduce the magnitude and duration of any failures and are, therefore, captured in the aforementioned definition.

Sustainability is typically expressed as a set of socially derived goals or capitals to be maintained or even enhanced for future generations (Jenks and Jones 2010). It is defined here as “the degree to which the system maintains levels of service in the long‐term whilst maximising social, economic and environmental goals” (Butler et al. 2014). This reflects the conventional “three pillars” of sustainability, but it must be noted that the goals can be conflicting and trade‐offs may be required (Matthew and Hammill 2009). Global sustainability goals relevant to the water industry might include United Nations targets (United Nations 2016), such as access to safe and affordable drinking water for all (social and economic), restoration of water‐related ecosystems (environmental), strengthening participation of local communities in improving water management (social), and more efficient use of resources to decouple economic growth from environmental degradation (economic and environmental).

### Properties and performance

It is important to acknowledge the property/performance duality of the terms reliability, resilience and sustainability: the Definitions section specifies the performance required from a system (in relation to level of service provision) but not the properties of the system required to deliver it. To be classified as reliable, resilient, or sustainable, specified performance goals must be met, and this may be achieved through manipulation of properties. Many different system properties may contribute to the reliability, resilience, or sustainability of a system, but any one property does not guarantee a certain level of performance. Increasing flexibility, diversity, and redundancy, for example, may be seen as methods by which resilience and/or sustainability may be enhanced (Holling 1996; Cabinet Office 2011). However, these are *properties* of a system, and their effect on *performance* of a system is uncertain. Connectivity is another system property that may be assumed to provide resilience (USEPA 2015); however, increased connectivity does not guarantee increased resilience because highly connected nodes are particularly vulnerable to a targeted attack (Albert et al. 2000). In this respect, continued service delivery could be viewed as an emergent feature of a complex system with multiple interacting properties, and as such, performance may be unknowable when the system is subject to exceptional conditions.

Properties and performance are often muddled, and this causes confusion. An example is the UK Cabinet Office (2011) advice to improve resilience by increasing resistance, reliability, redundancy, and response and recovery – an unspecified mix of performance measures (reliability and response and recovery [time]) and properties (resistance and redundancy). A second example is where technology is given a label that implies a particular performance, such as sustainable drainage systems (Fletcher et al. 2015). Although these systems are asserted to have sustainable properties, their performance may or may not be sustainable depending on the framings or indicators used. Linking properties to performance is an ongoing research endeavor (Mugume et al. 2015a).

Analysis of the levels of performance required for reliability, resilience and sustainability provides a basis through which they can be compared and their relationships explored:

- Reliability addresses performance within the design life of the system and during periods in which the required level of service is expected to be met (i.e., when subject to standard loading/in the absence of exceptional conditions). Reliability‐based design aims to provide fail‐safe performance, that is, avoid failure.
- Resilience relates to performance during the design life but addresses performance during periods in which the required level of service is not met (i.e., when subject to threats). Resilience‐based design aims to overcome failure and ensure that the system is *safe to fail*.
- Sustainability relates to the level of service provision (performance) and social, economic, and environmental consequences in the long term (i.e., up to and beyond design life). This encompasses all levels of performance, both above and below the required level of service.

Reliability, resilience and sustainability are seen as separate but linked entities where reliability is necessary but not sufficient for resilience, and resilience is necessary but not sufficient for sustainability (Scholz et al. 2012; Blockley et al. 2012; Pickett et al. 2014).

## Analyzing Reliability, Resilience and Sustainability

As noted previously, operationalizing these concepts is difficult because of the lack of a common approach to their analysis. In response to this, a framework has been developed that uses threats, systems, impacts, and consequences as starting points from which reliability, resilience and sustainability can be analyzed.

### Threats

A key facet of conventional analyses is the identification, characterization, and categorization of potential threats. A threat is defined here as “any event with the potential to reduce the degree to which the system delivers a defined level of service” and is a synonym for a wide variety of other terms used in literature, including hazard, event, perturbation, disturbance, shock, and crisis. These terms are commonly used in traditional risk management approaches, where the emphasis is on the probability of their occurrence and the resultant effects (Public Safety Canada 2012).

Threats can be categorized in a number of ways, and these will be context and/or system specific. One approach is to classify threats as either internal or external, depending on their origin (Jen 2003; Westrum 2006; Jansen et al. 2007; Lansey 2012 2012). Threats have also been classified based on their rate of change (i.e., chronic or acute) (Jansen et al. 2007; Hamilton 2009; Madni and Jackson 2009; Cimellaro et al. 2010).

**Figure 1:**
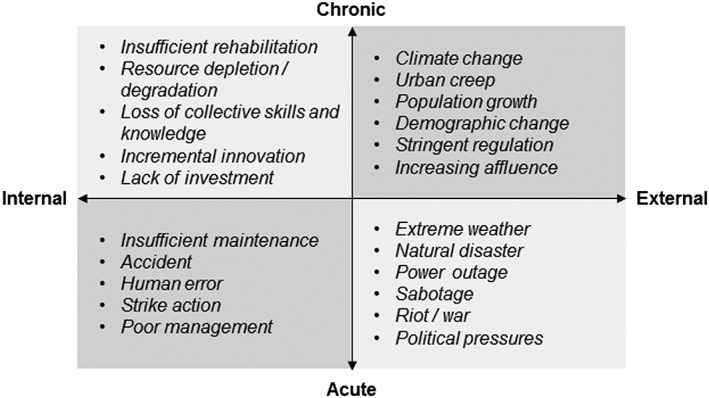
Threat categorization and examples.

In this framework, four threat subcategories are proposed, building on the work of Dawson et al. (2010). These can be presented as four quadrants – external–chronic, external–acute, internal–chronic, and internal–acute – as shown in Figure 1. Internal denotes a threat that originates with the water service provider or water infrastructure, such as a lack of investment or a poor maintenance regime, whereas the influence of any outside force, entity, or actor (e.g., the natural or build environment, water users, or water regulators) signifies an external threat. Chronic threats are those that occur gradually, for example, urban creep. These are typically expected and/or predictable. Acute threats, for example, natural disasters, happen quickly and are usually unexpected and/or unpredictable. Further examples are provided in the corresponding quadrants of Figure 1, but in practice, these should be updated according to the system under consideration and the needs of the user.

This categorization structure aims to support better identification of threats in a range of contexts by water service providers, policymakers, and regulators. Partitioning of the “threat space” may also facilitate a more targeted analysis approach and aid prioritization of interventions.

### Water system (middle states)

The water system can be considered a social–ecological–technical system, comprising natural, physical, organizational, and social systems (Newman et al. 2011; Hodbod and Adger 2014), and understanding and analysis of such a system is often a key component of efforts to enhance reliability, resilience and sustainability (e.g., Hamilton 2009).

An important concept related to the system is that of middle states (Johansson 2010): these occur as a result of threats and represent all the potential modes of failure for a given system. An example in a water distribution system would be pipe break, which could result from many different threats. However, while multiple threats can result in the same middle state, there are still many ways in which a system can fail, and it has been suggested that further work on failure modes could help with the identification of options that would improve resilience (Watts et al. 2012).

**Figure 2:**
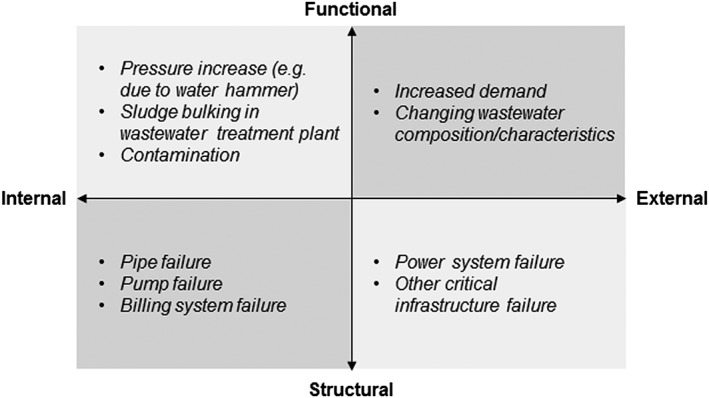
Water system middle states categorization and examples.

Middle states can be categorized using a four‐quadrant classification, as for threats. However, the temporality of change is not relevant here, and failure modes are instead classified as functional (operational) or structural. Example middle states in each category are shown in Figure 2.

### Level of service impacts

Threats, when made active, can have many effects and result in outcomes for a range of entities, either directly or indirectly. In the literature, the terms “impact” and “consequence” have commonly been used interchangeably to represent these effects, for example, in risk analysis studies and emergency planning (e.g., Cabinet Office 2011; IPCC 2012). To facilitate the decision making process in the development of interventions and enable a well‐defined analysis while avoiding confusion, the two terms are assigned specific meanings in this work and not used interchangeably.

Impacts are expressed at a system level and are the direct result of system failure. They are defined here as “the degree of non‐compliance with a defined level of service.” Required levels of service may be based on local‐level, national‐level, international‐level, or global‐level standards and are used by regulators to monitor the quality of service provided. The World Health Organization, European Union, United States, and Australia (among many others), for example, all specify acceptable limits for drinking water constituents such as ammonia, chloride, arsenic, and fecal coliform bacteria (United Nations 2007), and the World Health Organization provides minimum water quantity requirements (Howard and Bartram 2003): these represent required levels of service. At a smaller scale, discharge concentrations from an individual wastewater treatment plant, for example, may be considered measures of level of service, and concentrations in excess of those permitted by the local environmental protection agency represent noncompliance.

Previous resilience assessments have often measured performance using abstract indices (Ash and Newth 2007) or a more qualitative approach (Gupta 2007). By relating the impact directly to level of service measures used in water sector, the proposed framework aims to provide greater operational relevance.

**Figure 3:**
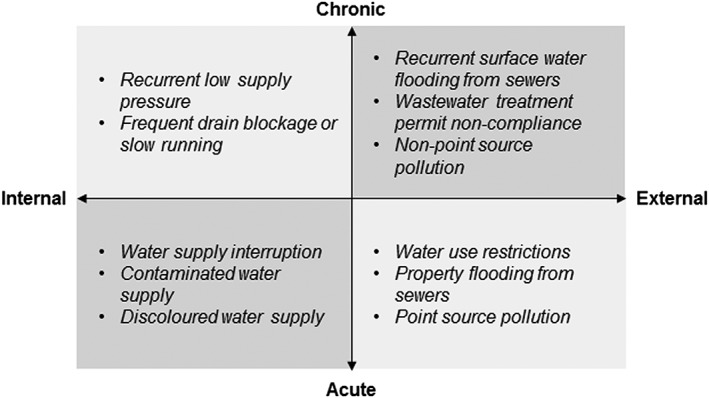
Impacts categorization and examples.

Impacts can also be mapped onto the four‐quadrant model presented previously, and examples are given in Figure 3.

### Societal, economic, and environmental consequences

Consequences are considered in this framework to be distinct from impacts and are defined as “any social, economic or environmental outcomes for a recipient due to the effects of non‐compliance with a level of service.” They are the direct result of impacts and are “recipient centric.” The social, economic, and environmental consequences correspond with the social, economic, and environmental dimensions (three pillars) of sustainability identified by the United Nations (1993) and incorporated in the Definitions section. Assessment of sustainability within this framework will, therefore, be based on analysis of consequences.

**Figure 4:**
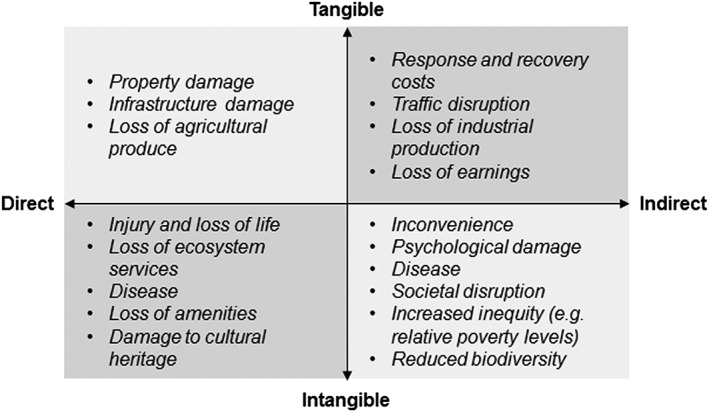
Consequences categorization and examples.

As with the other elements, consequences can be mapped onto a four‐quadrant model (Fig. 4), but different axes are required. Here, consequences are categorized based on their tangibility (tangible or non‐tangible) and directness (direct or indirect) (SCARM 2000). Loss of earnings, for example, is a tangible consequence of flooding (i.e., it can be quantified in monetary terms), whereas spread of disease is intangible; damage to property is a direct consequence of flooding, but reduced industrial production may be an indirect consequence. Identification of the scale of different consequences may also be beneficial when addressing community resilience, because communities comprise individuals with diverse attitudes, perceptions, and behaviors for whom responses and repercussions will differ (Barr et al. 2011).

## Interventions Framework

### Framework

**Figure 5:**
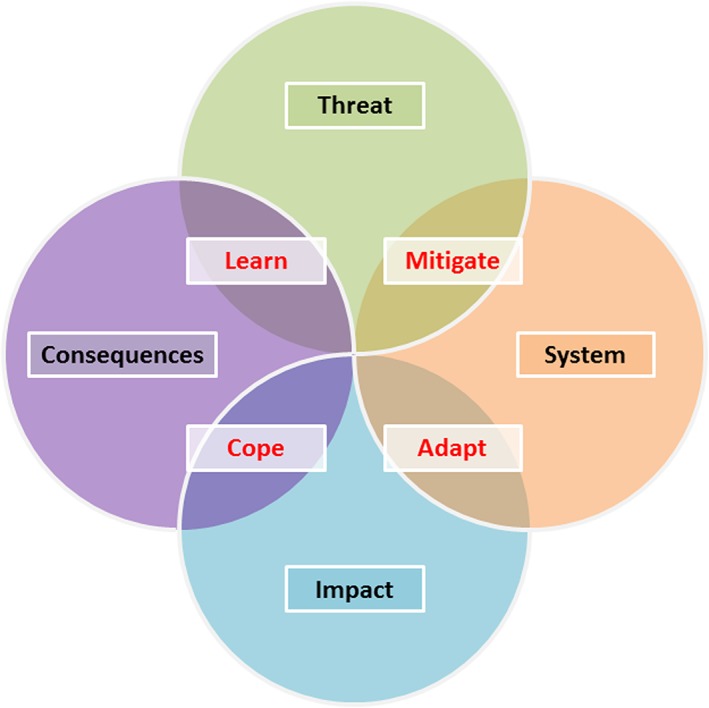
Intervention framework.

The aforementioned analysis elements are combined in the framework shown in Figure 5, which provides a diagrammatic representation of the relationship between threats and their social, economic, and environmental consequences. It also clarifies the role of the water system in mediating between threats and compliance with defined levels of service (impacts). Essentially, threats result in system failures (if mitigation measures are insufficient); system failures result in impacts (if adaptation measures are insufficient); impacts result in consequences (if coping measures are insufficient); and learning aims to embed new knowledge, thereby reducing consequences resulting from a threat.

While the framework has been developed in collaboration with UK‐based practitioners, challenges to water management are present worldwide in both high‐income and low‐income countries and at a range of scales, from local to global. The nature of these challenges and the intervention strategies used to address them may differ, but the framework is widely applicable and can be used with any combination of threats, systems, impacts, and consequences. Some consequences of flooding will differ in Bangladesh and England, for instance, but the conceptual relationships between threats, systems, impacts, and consequences presented in the framework would not change.

These concepts are elaborated in the following discussion.

Figure 5 is based on the framework proposed by Butler et al. (2014) but has been modified to incorporate an additional intervention and facilitate a “circular” analysis approach (discussed in the Interventions and Applying the Framework sections). It reflects elements of the socioeconomic driver–pressure–state–impact–response framework for reporting on environmental issues (EEA 1999) and disaster resilience frameworks (DFID 2011), which link multiple elements relating to disaster response. A key advantage of this framework, however, is that it enables opportunities for interventions to enhance resilience and sustainability to be identified at multiple stages in a clear and methodical manner. It also incorporates the idea promoted in the accident scenario “bow‐tie” approach (Hale and Heijer 2006) that analysis can be carried out from different directions but provides greater flexibility and more analysis options than the existing bow‐tie model. Lastly, it is simple, transferable between systems, and widely applicable.

### Interventions

Four types of intervention are identified in Figure 5: mitigation, adaptation, coping, and learning. All ultimately result in a reduction in negative consequences (or potentially even an increase in positive consequences) and are discussed in the following sections.

#### Mitigation

Mitigation addresses the link between threat and system and typically denotes long‐term actions to ameliorate threats that, although carried out locally, could have wider benefits (e.g., Klein et al. 2007). In this context, mitigation is defined as “any physical or non‐physical action taken to reduce the frequency, magnitude or duration of a threat.” Reducing greenhouse gas emissions would be an example of a mitigation measure that may be employed both locally and globally to reduce the magnitude of global warming.

Mitigation measures are specific to the threat and can, therefore, be mapped onto the same four‐quadrant model. Examples are given in Table 1. Selection of mitigation strategies will depend on the available technologies and resources (natural, human, and financial) available, as well as the priorities and objectives of the decision makers.

**Table 1:** Example mitigation techniques.

Quadrant	Threat	Mitigation measure
Internal–chronic	Insufficient rehabilitation	Accelerate asset replacement strategy
Internal–acute	Accidents	Develop safety culture
External–chronic	Urban creep	Enforce planning controls
External–acute	Extreme weather	Reduce greenhouse gas emissions of operations

Not all threats can be mitigated (e.g., unforeseeable threats), so while system failures can be reduced with the implementation of mitigation measures, they cannot be prevented entirely and further interventions are required to minimize negative consequences.

#### Adaptation

In this framework, adaptation measures are interventions that address the link between system and impact and deal with system failures that result from threats that cannot be mitigated. Definitions and applications of the term “adaptation” in the literature are varied, but adaptation is typically considered to entail targeted actions or adjustments carried out in a specific system in response to actual or anticipated threats in order to minimize failure consequences (e.g., Jones and Preston 2011; IPCC 2014). Given that “consequences” and “impacts” are distinguished in this work, however, adaptation is defined explicitly as “any action taken to modify specific properties of the water system to enhance its capability to maintain levels of service under varying conditions.” Such actions may be taken before, during, or after a disruptive event and can increase reliability, enhance resilience, and/or improve sustainability (Grothmann and Reusswig 2006). Adaptation measures are commonly discussed with respect to a specific threat – Adger et al. (2005), for example, discuss adaptations to climate change. It is argued here, however, that adaptation addresses the system failure – whether this is caused by climate change or another threat is to some extent irrelevant.

Adaptation measures each address a specific system failure mode and can, therefore, be mapped onto the same four‐quadrant model as the middle states. Examples in each quadrant are given in Table 2.

**Table 2:** Example adaptation techniques.

Quadrant	Middle state	Adaptation measure
Internal–functional	Sludge bulking	Operational modifications
Internal–structural	Pump failure	Provision of backup pumps
External–functional	Increased demand	Promotion of water saving technologies and use of reclaimed water
External–structural	Changing regulations	Provision of additional treatment/new technologies, for example, nutrient recovery

In practice, interventions may provide both mitigation and adaptation. Implementation of permeable paving, for example, may reduce urbanization effects (mitigate the threat) and also be a means of adapting to reduce excess storm water runoff entering the sewer system (a system failure mode that could result from multiple threats).

While adaptation measures can reduce the impact of system failure, it is not possible to adapt sufficiently to completely avoid all levels of service failures, and further intervention is required to address these.

#### Coping

Within the framework, coping addresses the link between impact and consequence. Typically, it has been seen as any response to threats and their impacts (Kabat et al. 2002), but it is defined more specifically in this work as “any preparation or action taken to reduce the frequency, magnitude or duration of the effects of an impact on a recipient.” Coping is often temporary and is actualized should existing mitigation and adaptation measures be insufficient to ensure compliance with required levels of service. Depending on the context and the recipient, coping strategies can range from provision of emergency supplies or protection provided by the water service provider through to support from external agencies or self‐protective behavior (Grothmann and Reusswig 2006).

As an example of coping at a property scale when subject to flooding, strategies could include purchase of household insurance to ensure that damage costs are reimbursed, installation of waterproof barriers on gates and doors, moving of valuables to upper floors, or relocation of occupants to temporary accommodation (Grothmann and Reusswig 2006). Coping strategies address specific consequences and, as with other interventions, can be mapped onto the corresponding four‐quadrant model. Further examples in each category are given in Table 3.

**Table 3:** Example coping techniques.

Quadrant	Consequence	Coping measure
Direct–tangible	Property damage	Temporarily relocate
Direct–intangible	Spread of disease	Boil water
Indirect–tangible	Response and recovery costs	Purchase buildings insurance
Indirect–intangible	Reduced biodiversity	Re‐introduce species

#### Learning

Negative consequences cannot be eliminated entirely by mitigation, adaptation, and coping. The final intervention, therefore, is learning, which is placed at the intersection of consequences and threats in the framework and defined as “embedding experiences and new knowledge in best practice.” Unlike the previous interventions, it need not address specific threats, impacts, or consequences and is relevant to all four‐quadrant models.

There are many approaches to learning, which can include learning from past events, developing pilot schemes to generate new knowledge for best practice, and learning from others. Good data collection and effective communication strategies can also facilitate learning. In all cases, it is important that lessons are learnt from both good and bad practices (Ferguson et al. 2013).

### Applying the framework

**Figure 6:**
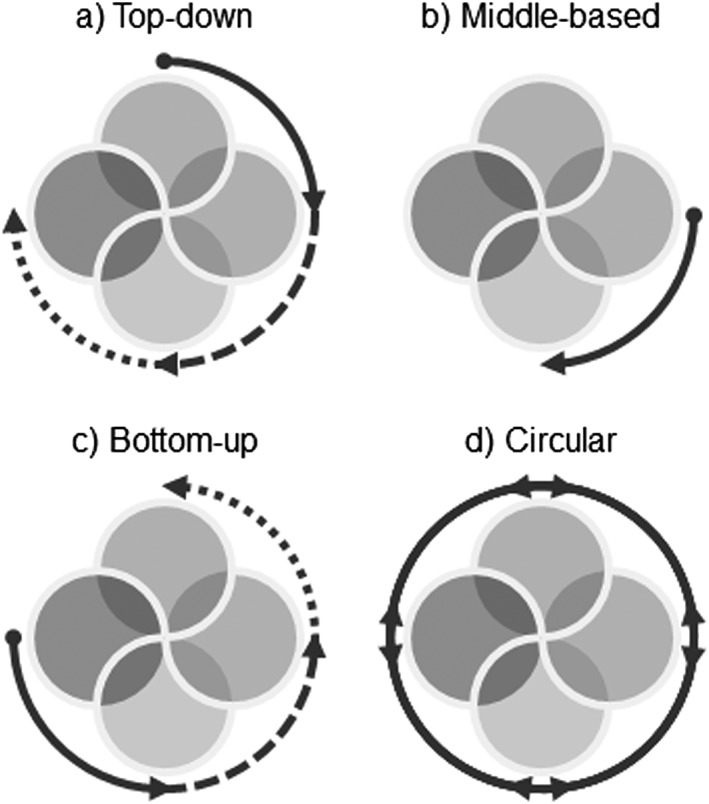
Analysis approaches. For interpretation of interlocking circles, refer to Figure 5.

The interventions framework presented facilitates analysis from different “directions”: top‐down, middle based, bottom‐up, and circular. These directions build upon the model A and model B planning approaches proposed by Geldof and Stahre (2006), where model A represents traditional planning approaches and model B represents a mode of thinking whereby the “water system” is considered as being inclusive of society. The four directions are presented visually in Figure 6 and explored in the following sections.

#### Top‐down

A top‐down analysis (Fig. 6a) is threat based and mitigation focussed and relies on identification of potential threats that may then be embedded in the planning process. Climate change, for example, is a widely recognized threat to water management and commonly considered when planning for the future. Top‐down methods move clockwise around the framework, typically from threat to impact or threat to consequence. Risk assessment, for example, typically uses a top‐down approach to evaluate the effects of a given threat (whether on level of service or society, economy, and the environment). However, even moving from threat to system can be useful for the development of mitigation measures.

This approach to water management represents the conventional planning process, where decisions are made centrally and system design decisions are assumed to deliver the required level of service under prevailing conditions. While it is recognized that this approach does not reflect the multiple feedbacks present in a social–ecological–technical system and may be inadequate for complex, adaptive systems (Geldof and Stahre 2006), it is still widely used and therefore worthy of discussion. A top‐down approach has been used in hydrological studies, catchment management, and integrated urban water management (Bai et al. 2009; Casal‐Campos et al. 2015; Hickel and Zhang 2006), for example, and also for risk management studies and emergency planning (Jansen et al. 2007; Cabinet Office 2011; Public Safety Canada 2012).

#### Middle based

In top‐down approaches, as typified by traditional risk analysis, emphasis is placed on defining and assessing the probability of a threat and then determining its effects. The difficulty with this approach is that each threat might have several different impacts, and indeed, different threats might have the same impact. Even more challenging can be the identification of all threats (Cabinet Office 2011) – unknown unknowns, for example, are the threats of most concern in resilience analysis but by definition cannot be identified. To overcome this, the framework lends itself to conceptualization of a middle‐based approach (Fig. 6b), which considers failures modes within the water system (middle states), their impacts, and the intersecting intervention of adaptation. Such an approach shifts the emphasis from identification and analysis of multiple threats to the more easily identifiable and measurable response of the level of service provision to system failure (i.e., middle states). The key benefit here is that multiple threats (including those that are unknown) that result in the same system failure mode can be addressed with a single analysis, thereby enabling a more comprehensive resilience assessment and improving the adaptation development process.

**Figure 7:**
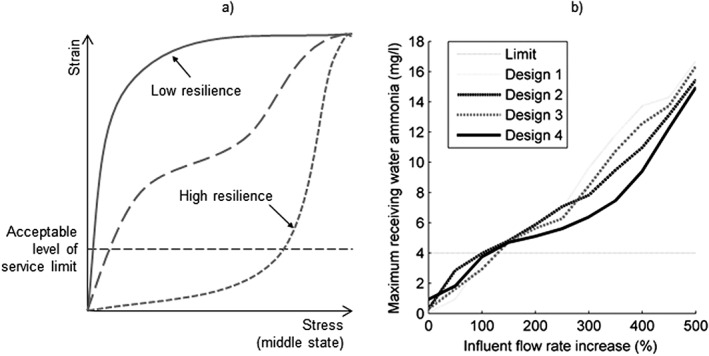
Middle state “stress–strain” curves for system designs with different levels of resilience to a given system failure mode (stress): (a) conceptual illustration and (b) example integrated urban wastewater system results.

Response curves of the form shown in Figure 7 demonstrate how a middle‐based approach may be used to support the development of resilient design and operational strategies. Figure 7a illustrates the concept, whereas Figure 7b shows example results for a range of integrated urban wastewater system design options. Each curve represents performance of a different system design under a range of stress magnitudes, where “stress” is a measure of the middle state (e.g., percentage increase in influent flow) and “strain” a measure of the impact (e.g., receiving water peak ammonia concentration). Note that the maximum stress considered represents an extreme case and is highly unlikely to occur in reality. All system designs in Figure 7a and b provide the required level of service under zero stress conditions (i.e., they are reliable), but their performance deteriorates as stress increases. The system with the smallest area under its response curve and above the acceptable level of service limit can be considered the most resilient to the specified stress, and the effects of any adaptation measures would be reflected by a change in the response curve shape.

#### Bottom‐up

The framework can also be used to facilitate a bottom‐up form of analysis (Fig. 6c), which focusses on increasing resilience in the face of threats, impacts, and uncertainties. This can also be expressed as a focus on reducing vulnerability because, although the precise definition is contested (Joakim et al. 2015), vulnerability is commonly interpreted as an antonym of resilience (Miller et al. 2010).

A bottom‐up analysis is conventionally consequence based and coping focussed, starting with identification of potential social, economic, or environmental consequences and progresses anticlockwise around the framework. For example, the United Nations Educational, Scientific, and Cultural Organization's evaluation of flood vulnerability incorporates social, economic, and environmental measures of susceptibility such as human health, access to water supply, income, unemployment, urban growth, and natural reserves (UNESCO‐IHE 2016). However, the analysis may also start at the system stage and proceed anticlockwise if assessing vulnerability not from a society/economy/environment perspective but from an infrastructure criticality perspective (e.g., Yazdani and Jeffrey 2012; Diao et al. 2014).

An advantage of this approach is that consequence analysis can be carried out without detailed assessment of threats or even impacts, instead focussing on how an individual, household, organization, or community would cope with the consequences of the (usually temporary) removal of a critical system or service (e.g., Pitt 2008; Wilby and Dessai 2010; Jones and Preston 2011).

#### Circular

The final analysis approach facilitated by this framework considers the threats, system, impact, and consequences as part of a circular arrangement (Fig. 6d), with a focus on learning. This addresses all components of the framework because, where mitigation, adaptation, and coping actions have been implemented as part of a comprehensive strategy, assessment of their efficacy is vital to inform learning and ensure that strategies, processes, and actions are updated (Pitt 2008; de Graaf et al. 2009; Haasnoot et al. 2013).

The following example demonstrates the circular nature of the framework. Climate change represents a threat, and there is a need to minimize its effects on urban flooding. Long‐term mitigation actions, such as greenhouse gas emission reduction, are in place at national government and international levels to reduce the threat. Because mitigation measures will not eliminate the threat, water service providers are implementing adaptations such as larger sewers and retention tanks to minimize the impact of increased flows (i.e., minimize flood magnitude). These adaptations will not entirely prevent flooding, and coping actions are necessary to minimize the consequences of any flood. These might include emergency planning and support at a local authority level and individual preparations at a household level. Despite these interventions, however, there will still be negative consequences from flooding, with inundation potentially resulting in damage to property and possessions and temporary loss of public services. Learning actions, therefore, would aim to intensify mitigation measures (e.g., incentivize green energy sources), re‐evaluate system adaptation approaches (e.g., improve flood warning accuracy), and revise coping strategies (e.g., update emergency plans with key knowledge generated by experiences before, during, and after the event at individual, community, and organizational levels), thereby reducing the negative consequences of a given threat in the next cycle.

This circular approach enables capacity to be built at a number of levels and for preparedness for and ability to respond to threats to be increased, resulting in improved resilience and sustainability. Coordinating the processes and assigning ownership of the key responsibilities present a challenge to its implementation, but ongoing research will further develop it and enhance its usefulness.

## Safe & Sure Water Management

The definitions, concepts, and interventions framework presented in this paper form the foundation of the Safe & SuRe (Reliable, Sustainable, and Resilient) approach to urban water management. This paper builds upon the Safe & SuRe ideas introduced and briefly discussed by Butler et al. (2014), providing an update on the concepts and directions taken in light of recent insights. This includes clarification and expansion of the concepts, enhancement of the interventions framework, and further work on the framework applications. Research under the Safe & SuRe project is being undertaken in collaboration with practitioners and policymakers, with partners including water companies, consultants, and government organizations (as recognized in the Acknowledgments section). Co‐creating the framework and corresponding quantitative analysis tools with multiple stakeholders is essential if the new water management paradigm developed is to be championed into practice – their knowledge and experience are proving invaluable.

A key element of the Safe & SuRe approach is recognizing and embracing the growing consensus among researchers and practitioners that it would be too expensive to apply the existing “fail‐safe” approach used within cities and the water sector to all future threats, even if it were possible (Ahern 2007). Fail‐safe is a “dangerous illusion,” which builds expectations that a system will *never* fail and service delivery will be maintained, an unrealistic expectation given that some threats are unknown and cannot be foreseen (Wharton 2015). Instead, an alternative “safe to fail” ethos (Holling 1996) is needed to manage contemporary change and uncertainty. This ethos acknowledges that systems will fail and arguably with increasing frequency but it is the (type of) failure that needs to be predicted and managed rather than the threat that causes the failure. Systems should be designed and operated, therefore, to *overcome* rather than *avoid* failure altogether.

Recent and ongoing work by the authors is beginning to incorporate and operationalize the Safe & SuRe water management thinking and approaches presented here. A top‐down approach, for example, as detailed in the Top‐down section, has been used by Casal‐Campos et al. (2015) to assess the social, economic, and environmental consequences of a range of catchment‐scale “green” and “gray” drainage strategies (adaptation interventions) under four future scenarios (threats), including climate change, urbanization, and population change. A regret‐based approach was applied to assess the relative performance of each strategy under the threats incorporated into each scenario. This was undertaken through comprehensive modeling of an integrated urban wastewater system case study and highlighted the lower regret of green strategies with respect to end‐of‐pipe gray alternatives.

The type of middle state‐based resilience analysis detailed in the Middle‐based section is now being explored in detail for various water systems, including by Mugume et al. (2015a, 2015b). Mugume et al. (2015b) used a middle‐based approach to systematically investigate the resilience of an urban drainage system in Kampala when subjected to a wide range of pipe failure scenarios (e.g., collapse and blockage). Pipe failure envelopes of the form shown in Figure 7 and a new resilience index that combines the failure magnitude and duration in a single metric were applied to a system in Kampala, Uganda, to quantify residual functionality of the system. Different adaptation strategies were evaluated, and it was concluded that, in this example, adding distributed storage provides greater improvement in resilience than increasing centralized storage. The results of such analysis can inform future implementation of adaptation measures and provide justification for decisions.

A bottom‐up approach, as detailed in the Bottom‐up section, has been used by Bryan et al. (2015 2015) to examine the role of coping interventions and the applicability of protection motivation theory in the development of coping indices. The study focussed on the consequences of flooding and drought for a local community in Exeter, England, and, through the use of threat and coping appraisals, provided a greater understanding of the issues that must be addressed to improve resilience. It was highlighted, for example, that risk perceptions are low because of unawareness or denial; the consequences of extreme events are not easily understood; the majority of residents have not considered their coping capacities; and there is an over‐reliance on centralized mitigation and adaptation interventions such as flood relief channels or provision of additional water resources.

## Conclusions

This paper has presented definitions, concepts, interventions, and a framework that contribute to Safe & SuRe water management. The framework connects global challenges relating to climate change, energy, food production, agriculture, and health, all of which may pose a threat to water management and/or be a consequence of water system failure. It can also be generalized and applied more widely.

Use of the terms “reliability,” “resilience,” and “sustainability,” as well as related concepts such as “threats,” “impacts,” and “consequences,” is often contested, with many similar terms meaning subtly different things. The Safe & SuRe concept provides an overarching framework with clear definition and clarity of terminology throughout. This clarity enables its use globally and at a range of different scales by academics, practitioners, and policymakers alike.

The disparate themes of reliability, resilience and sustainability are integrated, and their relationships in terms of properties and performance are explored. An important concept is that the properties of a reliable/resilient/sustainable system must be distinguished from the performance: to be classified as reliable/resilient/sustainable, a specified performance objective must be met – modification of system properties is the method by which this performance requirement is achieved.

A framework has been presented, which logically relates emerging threats, the intervening water system (in its broadest sense), the impacts on system performance (expressed as levels of service), and the social, economic, and environmental consequences of level of service failure. A key merit of this format is that it graphically illustrates the different types of interventions that may be used and how they relate to other components in the framework, providing greater clarity to decision makers and enabling better informed choices to be made.

The framework can also be used flexibly to facilitate analysis from different directions and support the development of strategies to increase resilience and improve sustainability while ensuring reliable provision of services. This has been demonstrated with a number of model‐based examples, and the potential benefits of a middle‐based approach in particular have been highlighted. Implementing and evaluating interventions developed in practice represent a topic for future work.
